# P24 A case of mycoplasma-induced rhabdomyolysis

**DOI:** 10.1093/rap/rkae117.055

**Published:** 2024-11-05

**Authors:** Jessica Chan, Kathryn Biddle, Adam Gray, Michelle Fernando

**Affiliations:** Guy’s and St Thomas’ NHS Foundation Trust, London, United Kingdom; Guy’s and St Thomas’ NHS Foundation Trust, London, United Kingdom; Guy’s and St Thomas’ NHS Foundation Trust, London, United Kingdom; Guy’s and St Thomas’ NHS Foundation Trust, London, United Kingdom

## Abstract

**Introduction:**

*Mycoplasma pneumoniae* is an intra-cellular bacteria that can cause atypical pneumonia, often in four-yearly cyclic epidemics. Extrapulmonary disease occurs in 5-10% of cases and includes musculoskeletal, haematological, dermatological and cardiac manifestations. Rhabdomyolysis is a rare musculoskeletal manifestation of *Mycoplasma* infection with only a handful of reported cases worldwide.

We present a case of rhabdomyolysis in a young man who presented to hospital with sudden onset of bilateral thigh pain, weakness and dark urine on a background of new cough, sore throat, and fever. Polymerase chain reaction (PCR) testing of an upper respiratory tract sample was positive for *M. pneumoniae*.

**Case description:**

A healthy European 16-year-old male student attended the emergency department with sudden-onset bilateral thigh pain, weakness and dark urine. He had a one day history of fever, cough and sore throat. He denied recent strenuous exercise or trauma. Systems review was negative for symptoms suggestive of an underlying connective tissue disease. He was not taking any regular medication. He did not drink alcohol, smoke tobacco, or use illicit drugs. Over the previous six months there had been an increased rate of detection of *M. pneumoniae* both in the UK and across Europe.

On examination, he was febrile (38.3 degrees C) and tachycardic (139 bpm). There was no rash or skin changes suggestive of dermatomyositis. His chest was clear. Power was graded 4/5 on bilateral hip flexion, knee flexion/extension, and neck flexion. Power in his upper limbs and other lower limb muscle groups was normal.

His C-reactive protein (CRP) was elevated at 56 mg/L, as was his creatine kinase (CK) at 86,309 units/L, and alanine transaminase (ALT) 475 units/L. His white cell count was elevated at 13.2x10*9, neutrophil count 10.07x10*9 and creatinine 91umol/L. His urine dipstick showed haematuria. There was no consolidation on chest radiography. The working diagnosis was infection-induced rhabdomyolysis and he was treated with intravenous fluids.

He responded well to intravenous rehydration with resolving pain and improving muscle strength. On day two, his CRP peaked at 137 mg/L and CK at 109,354 units/L. On day three, his ALT peaked at 847 units/L and a nose/throat swab tested positive by PCR for *M. pneumoniae*. He was initiated on a course of azithromycin. His fever resolved and his muscle power returned to normal (day four). Subsequent blood tests showed declining CK levels, falling to 18,945 units/L on day five. Myositis-specific antibodies were negative.

**Discussion:**

*M. pneumoniae*, a small, intracellular bacteria without a cell wall is known to cause both upper and lower respiratory tract infections, including “atypical pneumonia” – a historical definition of pneumonia with negative sputum culture and non-response to penicillin or sulfonamide treatment. Unusually, *M. pneumoniae* also causes extrapulmonary manifestations most commonly dermatological (e.g. Stevens-Johnson syndrome), neurological (e.g. encephalitis), and haematological (e.g. haemolytic anaemia). The pathophysiology of extrapulmonary manifestations is poorly understood with proposed mechanisms including direct bacterial infection and molecular mimicry.

Rhabdomyolysis, the rapid breakdown of skeletal muscle, commonly presents with a triad of muscle pain, muscle weakness and dark “coca-cola” urine (myoglobinuria). Urine dipstick is commonly positive for red cells with an absence of erythrocytes on microscopy indicating myoglobinuria. Blood tests demonstrate elevated muscles enzymes including CK, ALT, AST.

Common causes of rhabdomyolysis include trauma, strenuous exercise, medications (e.g. statins) and illicit drugs (e.g. cocaine). Viral and bacterial infections are recognised triggers, with published reports of rhabdomyolysis secondary to COVID-19, influenza viruses and HIV among others. In this case, a thorough history, examination and laboratory features pointed towards an infectious trigger.

*M. pneumoniae*-associated rhabdomyolysis is rare, with less than 20 published cases worldwide. The management of rhabdomyolysis includes supportive care such as analgesia and fluid resuscitation as well as the underlying cause. The benefit of antibiotic treatment is unclear, but is generally recommended.

**Key learning points:**

• *Mycoplasma pneumoniae* is a common bacterial infection and cause of community acquired pneumonia (CAP). It is estimated to cause up to 7% of cases of pneumonia in adults but accounts for up to 40% of pneumonia in children. It typically occurs in four-yearly cyclic epidemics, possibly due to changes in herd immunity.

• The presentation of mycoplasma infection is highly variable, ranging from asymptomatic infection, to CAP, to diverse extrapulmonary manifestations. From a rheumatological perspective, mycoplasma can present with arthritis, vasculitis, or rhabdomyolysis. It is important to recognise that these manifestations are infection-driven in order to prevent unnecessary investigations. As is in this case, mycoplasma-associated rhabdomyolysis can occur in the absence of pneumonia.

• Early recognition of rhabdomyolysis with a focused history and prompt treatment are important to prevent complications, such as acute renal injury, cardiac arrhythmias, and even death. Diagnosis of *M. pneumoniae* is most commonly made through PCR on upper and lower respiratory tract samples. Mycoplasma serology, which was previously widely used, has been phased out due to superior diagnostic accuracy of PCR testing. When treated promptly, prognosis is good and patients can quickly return to their baseline function.

• Mycoplasma pneumonia is generally self-limiting and rarely life-threatening. The duration of extrapulmonary manifestations, which are often immune-mediated, is not well established. Macrolides (e.g. azithromycin) are the antimicrobial agents of choice for *M. pneumoniae* infection. *M. pneumoniae* species lack a cell wall and are therefore intrinsically resistant to beta-lactam and glycopeptide antimicrobial agents. Reports of macrolide-resistant *M. pneumoniae* are increasing and alternatives such as tetracyclines and fluoroquinolones should be considered in this circumstance.

• In conclusion, infection-related rhabdomyolysis should be considered in patients with a compatible history. In these patients, it is important to perform a respiratory sample PCR alongside testing for other infections as directed by clinical suspicion.

